# Dietary Habits in Patients with Chronic Spontaneous Urticaria: Evaluation of Food as Trigger of Symptoms Exacerbation

**DOI:** 10.1155/2018/6703052

**Published:** 2018-06-19

**Authors:** Jorge Sánchez, Andres Sánchez, Ricardo Cardona

**Affiliations:** ^1^Group of Clinical and Experimental Allergy, IPS Universitaria, University of Antioquia, Medellin, Colombia; ^2^Foundation for the Development of Medical and Biological Sciences (FUNDEMEB), Cartagena, Colombia; ^3^Faculty of Medicine, Corporation University Rafael Nunez, Cartagena, Colombia

## Abstract

**Background:**

Many patients with chronic spontaneous urticaria (CSU) identify different foods as triggers of their symptoms and frequently make dietary restrictions without enough information.

**Objective:**

To explore the diet habits of CSU patients and estimate the clinical impact of the foods most frequently reported to be suspect.

**Methodology:**

Patients were interrogated about their clinical history of urticaria. Skin prick test and sIgE serum were done for most frequently reported foods by patients. Food challenge test was also performed. A group of healthy subjects was included to compare the dietary habits and the results of the diagnostic tests.

**Results:**

Patients with CSU (n 245) and healthy (n 127) subjects were included. 164 (66%) subjects from CSU group and 31 (24%) from the control group reported at least one adverse reaction with foods. Food IgE sensitization was similar in both groups (17.5% versus 16.5%, respectively). 410 food challenge tests in 164 CSU patients and 38 in 38 control subjects were performed. 1.2% in CSU group and 0.7% in control group had a positive oral challenge test.

**Conclusion:**

Despite the high frequency of self-report by patients, foods are uncommon triggers of CSU. Nevertheless, food challenge tests have to be offered early during medical evaluation to avoid unnecessary restrictions.

## 1. Introduction

Urticaria is a common cutaneous disease, where the chronic form affects around 1% of general population and has an important impact in the quality of life. Chronic spontaneous urticaria (CSU) can appear at any moment and for that reason patients associate foods, drugs, and different activities as possible triggers of their exacerbations [1, 2]. Usually, patients avoid the suspicious food, and this action has implications in their diet as well as personal and social life. In acute urticaria, food may play a causal role in some patients, but in chronic forms the role of food as a cause or trigger of CSU is not so clear.

Some studies have evaluated how often the triggers considered by the patient actually are associated with their symptoms. In a previous study, we observed that the prevalence of inducible urticaria self-reported was 75%, but the prevalence based on positive challenge tests was only 36%, indicating that a high number of patients did unnecessary restrictions [3]. Hsu ML et al. [4], observed that 32% of patients with chronic urticaria self-reported food as a possible trigger of urticaria, but after one month a restrictive diet was ineffective in 82.9% of patients. These results suggested that a self-reported evaluation is not adequate for studying some triggers of CSU. Furthermore, the GA^2^LEN/WAO/EAACI guidelines do not recommend any particular dietary restrictions for urticaria patients, except when a clear relationship is demonstrated [5]. However, to demonstrate “a clear relationship”, challenge tests are required and most studies evaluating the prevalence of food as a cause or trigger of chronic urticaria do not include this diagnostic test.

In this study, we evaluated the role of foods as triggers of urticaria in CSU patients, taking into account not only self-reported information, but also challenge test. As a secondary objective, we evaluated the frequency of IgE sensitization to the most common reported foods reported and their possible association as causal mechanism.

## 2. Methodology

### 2.1. General Characteristics

Based on a previously described cohort (URTICA project, ClinicalTrials.gov Identifier: NCT01940393) [6], we collected data from patients older than 12 years diagnosed with CSU, which was defined as the recurrence of hives for at least 6 weeks, in whom the diagnosis had been made by an allergist or a dermatologist. The severity of the disease and quality of life were evaluated with UAS (Urticaria Activity Score) and DLQI (Dermatology Life Quality Index), respectively. The exclusion criteria included the following: use of omalizumab; systemic disease associated with the hives; use of systemic corticosteroids for three weeks before recruitment; immunodeficiency, dermatitis, and/or any other disease that could alter the oral challenge or skin test results. Patients using antihistamines were included, but they had to be suspended at least 4 days before the challenge test.

The control group consisted of healthy subjects without urticaria to compare the prevalence results of the self-reports, the frequency of IgE sensitization (atopy), and the challenge test results from the CSU group. The control group consisted of people older than 12 years, with no history of chronic urticaria in the last two years. Prior to enrollment a physician evaluated each person in the control group.

### 2.2. Study Design

The study aim was to explore the diet habits of CSU patients and estimate the clinical impact of the foods most frequently reported as suspect. To reach this goal, we evaluated the role of foods as triggers of urticaria exacerbation in patients with CSU using self-reported data, IgE sensitization, and challenge tests. All subjects in the CSU and control group filled out a questionnaire where they identified possible previous acute reactions to any food.

Skin prick test (SPT) and measurement of specific IgE (sIgE) by immunofluorescence were performed to 10 foods (beef, pork, chicken, shrimp, fish, milk, egg, strawberries, soybeans, and wheat) in all the subjects in both groups. These ten foods were chosen based on the results of the questionnaire about the most frequent foods associated with urticaria exacerbation in the same population. Additional foods were tested in those patients with self-reported reactions with other foods. Those foods that did not have standardized extract (e.g., sauces, “spicy foods”) were directly tested by prick-by-prick and/or by oral challenge test.

The oral challenges were made with foods that each patient reported as suspect. Also, we did food challenge test to those foods that were not reported by patients but were positive in SPT or IgE serum.

### 2.3. IgE Sensitization Assessment

***Skin tests:*** The IgE sensitization to beef, pork, chicken, shrimp, fish, milk, egg, strawberries, soybeans, and wheat was assessed by skin prick tests (SPT) according to international guidelines [7, 8]. Sensitization to mites (D. pteronyssinus, D. farinae, and B. tropicalis) and pets' dander (Cat and Dog) was also investigated. In patients with other suspicious foods than those tested, additional tests were performed with it or them.

***Detection of serum IgE:*** Total and sIgE levels were measured in the serum using the ImmunoCAP 100 instrument (Pharmacia Diagnostic AB/Thermo Fisher, Uppsala, Sweden) according to the manufacturer's instructions. Results greater than 0.35kU_A_/L for sIgE were considered positive.

### 2.4. Food Challenge Test

Patients blinded placebo controlled food challenge tests with fresh foods were performed using another food that the patient tolerated to camouflage the taste. Those patients using daily antihistamine have to suspend it for at least 4 days before the challenge test. When patients had exacerbation before the challenge test, they could go to medical office or send a photographic register to be evaluated by their medical doctor and define if they required antihistamines or not. If patients required antihistamines, a new appointment for challenge test was offered.

Patients received a portion equivalent to the expected daily intake of the food investigated [9]. Foods for challenge test were selected according to the clinical history and self-report of the patients. We also did a challenge test for those foods with a positive sensitization test (SPT or IgE serum), independently of being reported as suspicious. In patients with self-reported food trigger but negative sIgE and SPT, challenge tests were made by administering the total food serving, divided into two portions separated from one another by one hour (10% and 90% of the total serving to be administered). In cases of positive IgE and a clinical history of reaction, food administration was divided into four portions (10%, 20%, 30%, and 40%). The evaluation period after the challenge was four hours and the patients were also instructed to give notice in case of late reactions. A challenge test was considered positive when the patient showed hives or angioedema during the evaluation period. Other symptoms like wheezing, diarrhea, and vomiting were also indicative of a positive test but were recorded separately if urticaria symptoms were no present.

The oral challenge was contraindicated for those patients with a clear clinical history of anaphylactic reaction in the last 12 months within less than an hour after ingesting the suspect food and with a positive SPT or serum sIgE.

### 2.5. Ethical Considerations

Institutional Review Board approved this study. The work description was carried out in accordance with the Code of Ethics of the World Medical Association (Declaration of Helsinki) for experiments involving humans: Uniform. Informed consent was obtained from each subject.

### 2.6. Statistical Analyses

Statistical analyses were performed using IBM SPSS Statistics for Windows, Version 21.0 program (IBM Corp, Armonk, New York). The mean and SDs were reported for descriptive variables. Differences between proportions were analyzed using the Pearson chi-square test.

Univariate analysis based on logistic regression was performed for categorical variables to assess the relationship between exposure and outcome (e.g., food sensitization and positive challenge or self-report). A* p* value < 0.05 was considered statistically significant.

## 3. Results

### 3.1. General Characteristics

A total of 245 patients with CSU (CSU group) and 127 healthy subjects (control group) participated in this study (Table 1). IgE sensitization and asthma were significantly more frequent in patients with CSU than in the control group (p < 0.05). No other differences regarding general characteristics were observed between the CSU group and the control group.

### 3.2. Self-Reported Food Triggered Exacerbations

**Figure 1:**
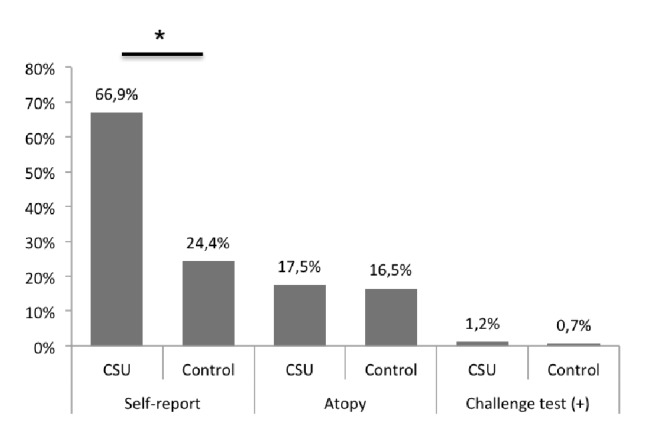
**Self-report, sensitization, and challenge test in CSU and control group.** Frequency of food sensitization and food trigger by self-report and challenge test to any food. Prevalence of Self-report, sensitization, and positive challenge test. Challenge test was done on 164 patients with CSU and 38 control subjects. The prevalence was calculated for the total number of patients in each group (CSU group 245, control group 127).

**Figure 2:**
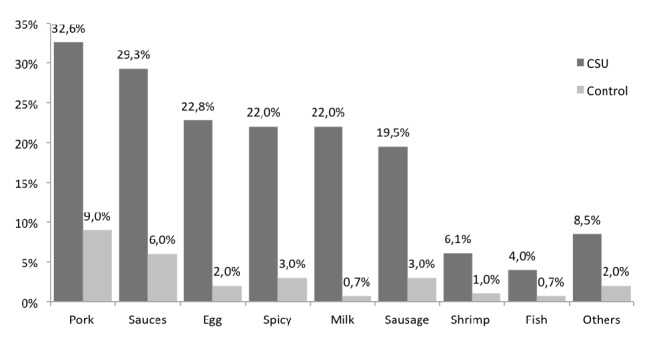
**Principal foods suspected by self-report.** Principal foods suspected by patients and control group.

**Figure 3:**
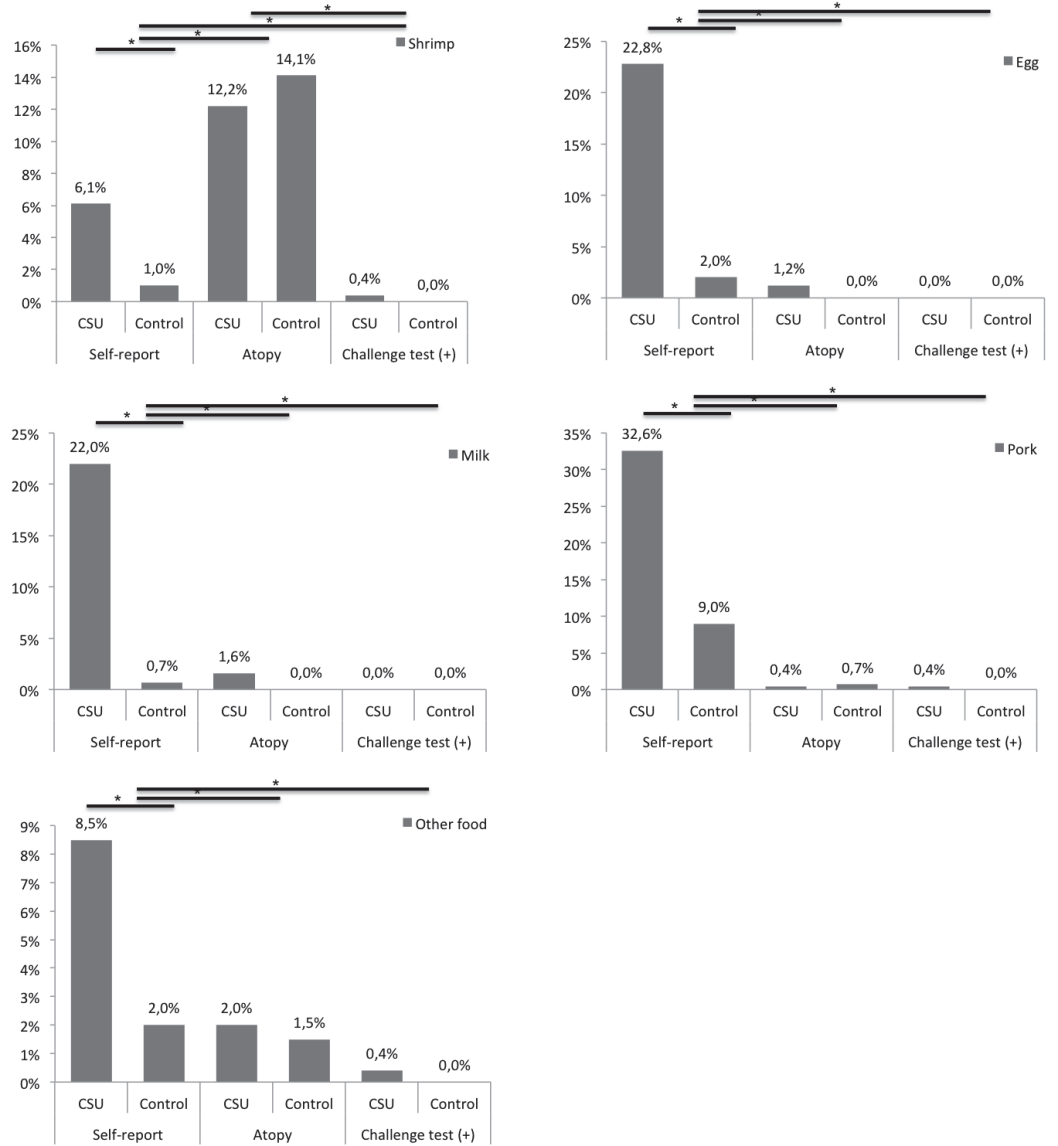
**Results of self-report, sensitization, and challenge test for the main suspect foods.** Percentages are based on the total number of patients with CSU or control group. ^*∗*^*p* <0.01.

One hundred sixty-four (66%) subjects from the CSU group and 31 (24%) from the control group reported at least one reaction with some food (p <0.01) (Figure 1) and 92% of them make a dietary restriction. In the group with urticaria, the patients self-reported an exacerbation of urticaria with these foods, while in the control group the symptoms that were self-reported were mainly cutaneous type pruritus, but 50% also reported erythema and hives. According to the reports of patients and control subjects, the primary food or food products suspected of causing reactions were pork and sauces for both groups (Figure 2). At least two foods were suspected of causing reaction in 40% of the CSU and 12 of the control group. Self-reported exacerbations were always higher than sensitization or positive challenge test except for shrimp (Figure 3).

### 3.3. Sensitization Evaluation

Food sensitization was similar in both groups (17.5% versus 16.5%, respectively) (Figure 1). There were no significant differences regarding SPT or serum sIgE for any food. Also, there were no significant differences between sensitization and challenge tests results except for shrimp (*p* <0.01) (Figure 3). Self-report of reactions to shrimp was less than 10% (Figure 2), but IgE sensitization was the highest among foods tested in CSU group (12.2%) and in control group (14.1%) (Figure 3).

Self-reported reaction to pork was 32% in CSU group and 9% in control group, but sensitization to pork was present in only one patient. A group of 21 patients (8.5%) reported other foods or food products as a potential for their urticaria: 5 had positive skin tests, which represents 23% of patients with self-reported exacerbations to foods but only 2% of the total of patients in the CSU group. One of them had a positive challenge test with mustard. IgE sensitization and oral challenge with beef, chicken, fish, strawberries, soybeans, and wheat were negative in all subjects from CSU and control groups.

### 3.4. Oral Challenge Test: Relationship with IgE Sensitization and Self-Reported Triggers

A total of 448 food challenge tests were made: 410 in 164 patients and 38 in 38 control subjects, respectively. Three patients in CSU group (1 shrimp, 1 pork, 1 pineapple) with negative sensitization tests (SPT and IgE) had positive challenge tests, representing 1.2% of the patients from the CSU group (Figure 3). IgE sensitization to shrimp was frequent in both groups, but the only patient with a positive challenge test result had negative SPT and serum IgE. Despite the high frequency of self-reported reactions with pork, only one subject (0.7%) in the control group had a positive test. This patient had a very suggestive clinical history of anaphylaxis in the last six months, so it was considered as a positive outpatient challenge and confirmation was not required for an additional challenge test.

None of the patients with self-reported exacerbations with egg, milk, sauces, spicy food, or fish in any of the groups had a positive challenge test. Of the 21 patients who reported reactions to other foods, one was sensitized and had a positive challenge test with mustard. There were no patients with positive sensitization tests or challenge test for other foods like sauces (tested in 48 from the CSU group and 4 from the control group), spicy foods (n= 54 and 4, respectively), fish (n=10 and 1, respectively), or any other food tested. None of the subjects had a positive placebo reaction. During the administration of the placebo, some subjects in the urticaria group (n 8) and the control group (n 4) manifested itching; however, none presented objective reactions, so the challenge continued with food being tolerated in all cases.

### 3.5. Follow-Up

Patients with a negative challenge were informed that they could consume the tested food and six months after the challenge, they were questioned about outpatient consumption. Sixty-four percent of the patients had consumed the food again and only 2 patients reported having a reaction of pruritus without hives or angioedema. Among the 36% of the patients who reported not having consumed the food, 18% did not do it out of fear, 10% did not have the opportunity to consume it again, and 8% did not want it because they did not like its taste.

## 4. Discussion

In CSU it is common for patients to associate the onset of symptoms with different activities [3], medications [1], or foods [2, 10] that they were performing or consuming near the time of the reaction. Similarly to what we had previously reported for inducible urticaria [3], in this study, we found that in more than 95% of patients with self-reported foods reactions the food was not related to the onset of symptoms. Additionally, over 80% of these patients were carrying out unnecessary dietary restrictions that might be detrimental to their health.

Most of the guidelines discourage food as a cause of chronic urticaria; however, it is common that patients associate the consumption of some food with worsening of the condition or as the cause of it, yet few studies have been conducted to demonstrate or discard this association. The self-reported prevalence of food as a trigger of CSU is about 13 to 80% [10–13] with different foods considered suspect according to the diet and social customs of each population. In our study, we observed a four times higher self-report of food exacerbation in CSU patients than in the control group. Most of the suspected foods corresponded to common foods in the diet of the population like pork, egg, milk, or fish but there were negatives for sIgE and challenge in most of the cases, confirming the low relevance of foods in the CSU. We do not know for sure the reasons for the high self-report. One possible explanation is the lack of information about the disease that patients receive from primary care physicians, which reflects the need for greater disclosure of international urticarial guidelines among first-level care physicians, to avoid these errors. Another hypothesis that does not exclude the first one is that the patient identifies the last action he performs as a possible trigger for his illness, and because the urticaria has a spontaneous appearance, it can frequently occur around the meals.

Similar to our results, some studies using diet restrictions have shown that foods are not relevant in CSU and diet restrictions are ineffective [4, 10]. Nevertheless, other studies with diet restrictions [11–13] observed that 17% to 73% of patients with CSU achieve complete or significant remission of symptoms after restriction diets, which highlights the importance of the oral challenge to clear the patients' fears and show the real impact of foods in CSU [4, 14].

The use of the basophil activation test, SPT, and sIgE serum for the diagnosis of urticaria triggered by food had conflicting results [15, 16], most of them showing a low sensitivity and suggesting that these tests do not replace the food challenge. When we compared CSU and control group, we observed a similar frequency of food sensitization (17.5% versus 16.5%). However, in the self-report, most of the control subjects reported mild gastrointestinal and skin reactions, which were not reproduced in the provocation tests, which suggests that these reactions were not allergic. Compared to other foods, the higher IgE sensitization to shrimp found in our patients may be because of the fact that the sensitization to mites is prevalent in our environment and there is a high cross-reactivity between some proteins of these two species [17–19]. This is supported by the fact that 30% of patients with IgE sensitization to shrimp had not previously consumed it and practically all subjects with atopy to shrimp were also sensitized to mites. Because none of the patients with IgE sensitization to shrimp had a positive challenge to it, we can assume that in most cases this sensitization results from cross-reactivity and therefore is not clinically relevant for patients with urticaria. In Latin America and our population, sensitization to pollen grains is low (<10%) [20]. Therefore, despite the high frequency of atopy and allergic respiratory diseases in the two study groups, pollen-food allergy syndrome does not seem to be an aggravating factor in urticaria.

“Pseudoallergens” are substances that induce hypersensitive/intolerance reactions that are similar to true allergic reactions. They include food additives, vasoactive substances such as histamine, and some natural substances in fruits, vegetables, and spices. Some studies suggest that eliminating pseudoallergens from the diet can reduce symptom severity and improve patient quality of life. M Magerl et al., from 140 subjects, found that one of each three subjects made a substantial reduction in their medication without experiencing worse symptoms or quality of life after pseudoallergen-free diet [21]. In our study, we did not specifically evaluate food additives or “pseudoallergens”. It cannot be ruled out that in some patients foods with “pseudoallergens” may be the cause of urticaria or at least act as triggers. However, we tested the most frequent foods referred by patients and evaluated sIgE sensitization and their clinical relevance. Also, before entering the study, our patients had observed restriction diets on suspected foods without observing a significant improvement (data not shown).

One of the strengths of our study is that we conducted more than 400 challenge tests in 245 patients and 127 control subjects and we did a prospective follow-up to evaluate ambulatory tolerance to tested foods, which makes the results of the study quite reliable. During the follow-up, we observed that 18% of the patients did not eat the food out of fear even with a negative challenge test and medical support, which shows how this disease can have a significant impact on patients.

In conclusion, food challenge tests have to be offered early during the medical evaluation to avoid unnecessary avoidance of foods, as they are uncommon triggers of CSU.

## Figures and Tables

**Table 1:** Population characteristics.

**Characteristics**	**CSU group** **(n 245)**	**Control group** **(n 127)**	***p***
Age (y)	28 (14-50)	27 (15-55)	--
Age of onset (y)	25 (4-49)	NA	NA
Sex: female, n (%)	150 (61)	79 (62)	--
IgE sensitization^*∗*^, n (%)	105 (42)	37 (29)	0.04
Asthma, n (%)	36 (14)	5 (3)	0.05
Rhinitis n (%)	105 (42)	50 (39)	--
DLQI score, mean + SD	15 + 3	NA	NA
UAS, mean + SD	3 + 1	NA	NA
History of food urticaria (%)	164 (66)	31 (24)	0.03
History of Drug urticaria (%)	92 (37)	30 (23)	0.04

Values are presented as % or mean. DLQI: Dermatology Life Quality Index. UAS: Urticaria Activity Score. NA: not applicable. ^*∗*^Sensitization to mites or pets dander. --: > 0.05.

## Data Availability

Results of this study are supported in ClinicalTrials.gov Identifier: NCT01940393.
